# Vertical growth of Moso bamboo relies on local sensing and growth stress

**DOI:** 10.1093/plphys/kiaf452

**Published:** 2025-09-26

**Authors:** Nicola Trozzi

**Affiliations:** Assistant Features Editor, Plant Physiology, American Society of Plant Biologists; Department of Computational and Systems Biology, John Innes Centre, Norwich Research Park, Norwich NR4 7UH, United Kingdom; Department of Plant Molecular Biology, University of Lausanne, Lausanne CH-1015, Switzerland

Moso bamboo (*Phyllostachys edulis*), one of the tallest and fastest-growing grasses, produces hollow culms that can exceed 20 m in a single growing season (Chen et al. 2022). Such rapid elongation creates a structural challenge. A stem with a length:diameter ratio >100 should be prone to buckling or collapse. Yet bamboo remains upright, flexing under wind or snow but recovering its vertical posture with precision. Luan et al. (2025) show that this stability arises from a finely tuned system of local orientation sensing, internal growth stress, and structural reinforcement.

The researchers compared upright culms, naturally bent plants, and shoots grown at a 45° angle and then released them all to track posture changes over time. These artificially inclined shoots showed that each internode acts autonomously, bending upward to regain verticality and straightening once alignment is achieved. Some shoots even overshot the vertical position before settling into place and forming S-shaped curves, while others stabilized without oscillation. This segment-by-segment adjustment resembles proprioception in animals, where individual body parts sense and correct their own positions (Moulia et al. 2006; Bastien et al. 2013). Local sensing at each internode, rather than centralized control, provides an efficient mechanism for coordinating posture during the rapid production of dozens of internodes in succession.

Measurements of internal mechanical stress showed that posture control relies on growth stress, a system of tension and compression stored in culms during elongation (Boyd 1950; Alméras and Clair 2016; Fig.). Cutting grooves into culms and monitoring strain revealed longitudinal tensile stress along the surface of upright stems, giving them stiffness comparable to prestressed concrete. Bent and inclined culms displayed asymmetric stress patterns, with strong tension on the upper side of internodes and weaker tension or compression on the lower side. These gradients varied along the culm: they increased from base to tip in naturally bent plants and shifted across the bent-to-straightened transition in artificially inclined ones. This demonstrates that bamboo actively remodels mechanical stress to resist gravity and environmental forces. The system parallels reaction wood in trees, tension wood in angiosperms, and compression wood in conifers, yet bamboo achieves comparable stability (Huang et al. 2002).

**Figure.**
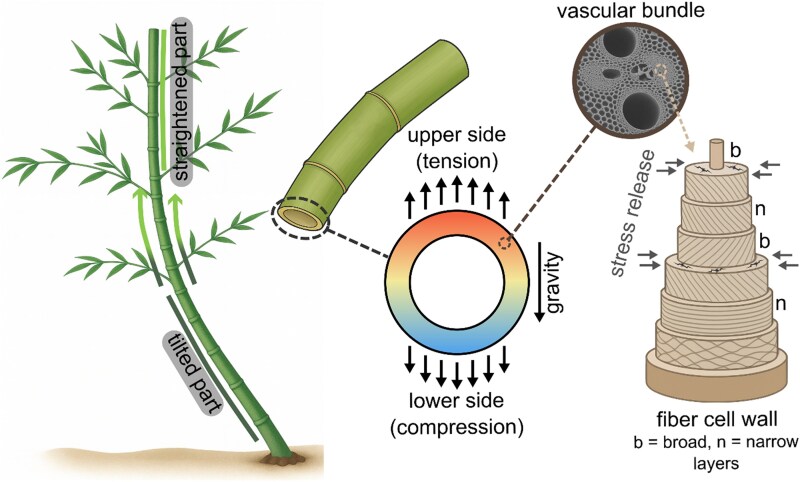
Multiscale posture control and stress regulation in Moso bamboo. Bamboo internodes sense their orientation and bend upward to correct posture, restoring vertical growth. Growth stress stored in culm tissues generates strong tensile stress on the upper side of bent stems and lower tension or compression on the lower side. The cross section shows this stress gradient, with arrows indicating tensile and compressive forces. A vascular bundle inset links culm anatomy to the fiber cell wall, where alternating broad (b) and narrow (n) layers form a concentric multilayered structure. Broad layers shrink more during stress release, producing cracks at their boundaries and storing tensile growth stress.

To understand how these stresses originate, the authors used atomic force microscopy to examine fiber cell walls at nanometer resolution. Stress release caused broad wall layers to shrink more than adjacent narrow layers, and separations sometimes formed at their boundaries. This layered wall structure appears to act as a spring system, storing tension during growth and retaining it after elongation ends (Yamamoto et al. 2002). Comparisons between polished and freshly cut surfaces revealed residual stress, confirming that the deformation was a direct response to stress release rather than an artifact of sample preparation. At the culm scale, wall thickness varied across cross sections, with thicker tissue present on the upper side of inclined stems, reinforcing regions subjected to the highest tensile load. These observations show how microscopic wall features support mechanical performance at the scale of the culm.

These findings establish bamboo as a model for studying biomechanics in fast-growing monocots. Understanding how plants generate and regulate internal stress has direct applications in materials science and agriculture, including the development of bamboo as a renewable construction resource. Future work on molecular signaling and wall biosynthetic pathways (Wang et al. 2024), as well as comparative studies of other tall grasses such as sugarcane and palms, may reveal whether similar strategies evolved independently and may broaden our understanding of plant structural adaptation.

Through the integration of whole plant imaging, direct mechanical measurements, and nanoscale microscopy, this study shows how bamboo achieves exceptional stability while growing at extraordinary speed. Stability arises from the interplay of local sensing, dynamic stress remodeling, and specialized wall architecture: each internode acts independently to adjust curvature, and fiber tissues generate tension that prestresses the stem, consistent with previous findings (Luan et al. 2024). Unlike woody plants that rely on secondary growth for reinforcement, bamboo reaches tree-like stature entirely through precise regulation of primary growth and stored stress distribution, functioning like a living suspension bridge under wind, gravity, and self-weight.

## Data Availability

No new data were generated or analysed in support of this research.
